# The diagnostic accuracy of a real-time optoelectronic device in cervical cancer screening: A PRISMA-compliant systematic review and meta-analysis

**DOI:** 10.1097/MD.0000000000011439

**Published:** 2018-07-20

**Authors:** Huixia Yang, Xinmiao Zhang, Zengping Hao

**Affiliations:** a Department of Obstetrics and Gynecology, Beijing Friendship Hospital, Capital Medical University, Beijing, China; b Department of Experimental Surgery-Cancer Metastasis, Medical Faculty Mannheim, University of Heidelberg, Mannheim, Germany.

**Keywords:** cervical cancer, real-time, screening

## Abstract

**Background:**

This study aimed to assess the diagnostic accuracy of a real-time optoelectronic device (TruScreen) for uterine cervical cancer screening.

**Methods:**

On the basis of Preferred Reporting Items for Systematic Reviews and Meta-analyses (the PRISMA statement) we performed this systematic review and meta-analysis. We searched PubMed, EMBASE, the Cochrane Library, CNKI, CBM, and WanFang Data using medical subject headings (MeSH) and text words. Title/abstract screening, full text check, data extraction, and methodological quality assessment (with the QUADAS-2 tool) were performed by 2 reviewers independently. The pooled sensitivity, specificity, positive likelihood ratio (PLR), negative likelihood ratio (NLR), diagnostic odds ratio (DOR), the summary receiver operator characteristic curve, and the area under the curve (AUC) were analyzed with Meta-DiSc software. Statistical heterogeneity was evaluated by Cochran's *Q* test and *I*^2^, meta-regression was conducted based on patient type, and the possibility of publication bias was evaluated using Deeks funnel plot in Stata software.

**Results:**

Of 293 publications, nine met our inclusion criteria. These studies included a total of 2730 patients and 567 cervical intraepithelial neoplasias. The pooled test characteristics for the TruScreen were as follows: sensitivity 76% (95% CI, 73–80%), specificity 69% (95% CI, 67%–71%), PLR 2.30 (95% CI, 1.59–3.33), and NLR 0.34 (95% CI, 0.23–0.51). The corresponding pooled DOR was 7.03 (95% CI, 3.40–14.55). The AUC was 0.7859 (*Q*^∗^ = 0.7236).

**Conclusion:**

The diagnostic accuracy of the TruScreen device is moderately good. The study findings are based on Chinese studies only and could not be generalized to other populations.

## 1 Introduction

Cervical cancer is one of the most common malignant tumors in women.^[1]^ The etiology of cervical cancer is clear. Methods of primary and secondary prevention of cervical cancer are well-understood. In some developed countries, nationwide cytological screening has dramatically reduced death rates from cervical cancer.^[2,3]^ In the developing world, however, absence of organized screening programs correlates with high cervical cancer death rates. Worldwide, the incidences of cervical cancer among different countries are significantly different. According to a survey by the International Agency of Research on Cancer (IARC), in 2012, about 85% of the cervical cancer occurred in less well-developed regions, accounting for 12% of female cancer. However, in developed countries, the incidence of cervical cancer accounted for only 3% of female cancer.^[1]^ In China, because of the large population and the unbalanced development of economics among different areas, the incidence of cervical cancer in different areas also show significant differences. For the western or remote mountainous areas, because of the lack of the economic, medical health facilities, and the medical staff, it is difficult to widely employ the cervical cancer screening strategy that is suitable for developed countries or areas. However, according to the recommendation of the World Health Organization (WHO), only when the cervical cancer screening coverage surpasses 80% could the aim of reducing the incidence and mortality of cervical cancer be achieved. It is more important to increase the coverage of cervical cancer screening than to increase screening frequency.^[4]^ Finding a cervical cancer screening technology that is simple, objective, instant, noninvasive, affordable, and could increase the cervical cancer screening coverage is a primary concern to gynecologists and policy makers in these areas.

A real-time optoelectronic device (TruScreen) was introduced in The Cervix (second edition) for use in cervical cancer screening.^[5]^ The device uses optical and electrical signals to analyze cervical tissues with a built-in algorithm. It is designed to be used without the need for aqueous acetic acid. The operator places the tip of the hand piece against the cervix, employing a single-use sensor. The operator pushes a button on the hand piece and the device prints out the screening result. The procedure takes approximately one minute to perform. The device is designed to be used by an operator without high levels of technical skill or training. This feature is particularly well-suited for areas where specialists are scarce and/or patient follow-up is difficult.

Results are reported as ”normal” (normal squamous epithelium, columnar epithelium, physiological metaplasia, or latent HPV-related changes) or “abnormal” for cervical intraepithelial neoplasia (CIN) I–III and invasive cervical carcinoma.^[6]^

A multi-center trial by Singer et al^[6]^ reported results from 671 patients in 10 centers. Sensitivity for pathologically proven CIN II/III by TruScreen was 70%. Shuyu Long et al^[7]^ reported results using this device in 181 patients. The sensitivity and specificity of the device for the detection of CIN was 67.4% and 68.1% respectively. Emre Özgg et al^[8]^ found that the sensitivity and specificity of the device for the detection of the CIN was 86.1% and 35% respectively. Together, these study findings do not constitute a consensus, as some of the sample sizes are small. Therefore, to provide conclusive evidence concerning the sensitivity and specificity of this real-time optoelectronic device for cervical cancer screening, we searched the literature for relevant studies and analyzed them quantitatively.

## 2 Methods

### 2.1 Search strategy

According to the Preferred Reporting Items for Systematic Reviews and Meta-analyses (PRISMA) framework,^[9]^ we developed this systematic review and diagnostic meta-analysis. We searched PubMed, EMBASE, the Cochrane Library, China National Knowledge Infrastructure (CNKI), China Biology Medicine disc (CBM), and WanFang Data from inception to November 2016. We combined medical subject headings (MeSH) and text words to identify eligible studies. The search strategy for PubMed was as follows: (((((cerv∗[Title/Abstract]) OR uter∗[Title/Abstract])) AND optoelectronic [Title/Abstract])) OR truscreen∗ OR truscan∗ OR polarprobe∗. To supplement the search, we scanned references of the retrieved studies. The search strategy for Embase was: truscreen∗ OR truscan∗ OR polarprobe∗ OR (optoelectronic:ti,ab AND (cerv∗:ti,ab OR uter∗:ti,ab)).

### 2.2 Inclusion and exclusion criteria

We screened articles according to the Cochrane methods group on systematic review of screening and diagnostic test recommend methods.^[10]^ Inclusion criteria were as follows: the purpose of the article was evaluation of the accuracy of TruScreen for cervical cancer screening; all the subjects included in the study underwent the index test, the gold standard test and been given a definitive diagnosis; use of the reference standard adopted by the 2003 WHO Classification of Tumors of the Breast and Female Genital Organs;^[11]^ results were reported in sufficient detail for reconstruction of 2 × 2 tables; publication in English or Chinese; studies involving ≥ 30 subjects; and the studies had to be published original articles. Exclusion criteria were as follows: no pathological diagnosis as gold standard; editorials, case reports, conference proceedings, and letters to editors; and insufficient data for describing or calculating sensitivity and specificity.

### 2.3 Data abstraction and quality assessment

Two reviewers performed data abstraction and quality assessment independently. All disagreements produced in the process were resolved by discussion with a third reviewer.

Data retrieved included: author, publication time, sample size, study design, reference standard, true-positives, false-positives, false-negatives, and true-negatives. We assessed the methodological quality of each study using the Quality Assessment of Diagnostic Accuracy Studies-2 (QUADAS-2) tool.^[12]^ The tool consists two parts: the risk of bias (four sections: patient selection, index test, reference standard, and flow and timing) and applicability concerns (three sections: patient selection, index test, and reference standard). Each item is rated “yes” (low risk of bias or good applicable) “no” (high risk of bias or bad applicability) or ”unclear” (insufficient data are reported). Revman 5.3 software was used in the process of methodological quality assessment.

### 2.4 Statistical analysis

Meta-DiSc 1.4 was used to analyze the estimates of pooled sensitivity, specificity, positive likelihood ratio (PLR), negative likelihood ratio (NLR), diagnostic odds ratio (DOR), and their 95% confidence intervals (CIs). We constructed the summary receiver operator characteristic (SROC) curve and calculated the area under the curve (AUC) and the *Q*^∗^ to evaluate the diagnostic accuracy of the TruScreen for cervical cancer screening. The AUC values of 0.5 to 0.7, 0.7 to 0.9, 0.9 to 1 indicating low, moderate and high diagnostic accuracy, respectively.^[13]^*Q*^∗^ is the point of SROC curve which is closest to the finest top left corner. When the AUC and the *Q*^∗^ were closed to 1, this would reveal a perfect test with 100 percent of specificity and sensitivity.^[14]^ We used Stata 14.0 to conduct the following analysis by a bivariate random-effect model. Heterogeneity was examined using Cochran's *Q* test and I-squared (*I*^2^) statistic. A *P* less than .1 for Cochran's *Q-*test and *I*^2^ greater than 50% for *I*^2^ statistics indicated significant heterogeneity.^[15]^ Regression analysis according to patient type was used for exploring the sources of heterogeneity. The possibility of publication bias was evaluated using the Deeks funnel plot. *P* < .05 was considered statistically significant.

In our article, ethical approval was not necessary, as this study is a meta-analysis, which is based on published data.

## 3 Results

### 3.1 Literature search overview

**Figure 1:**
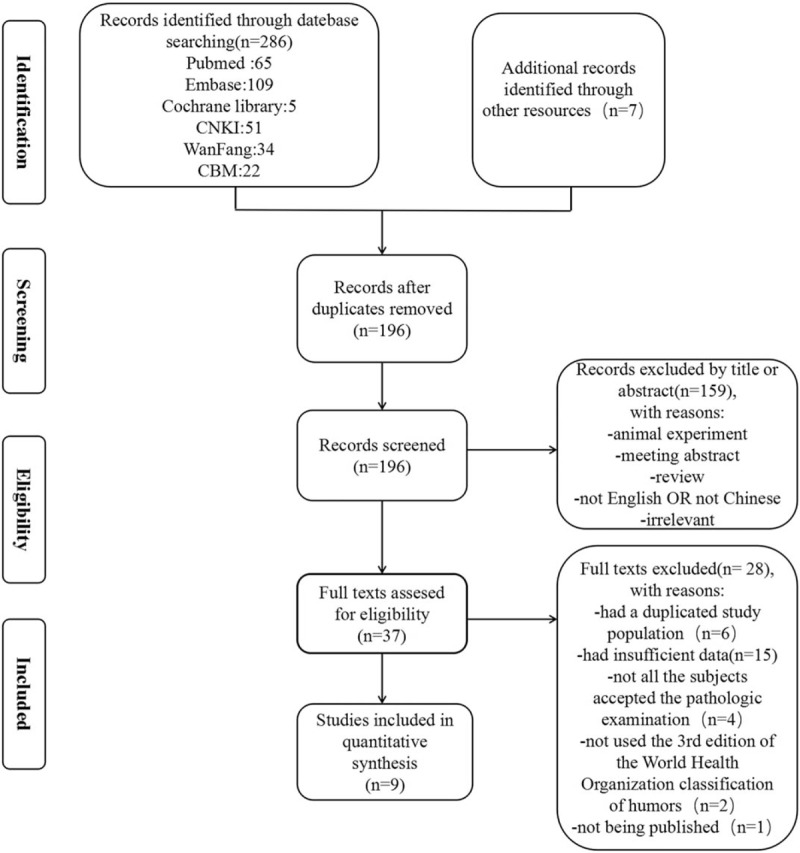
Flowchart of the study search.

The process of identifying qualified studies is shown in Figure 1. We identified 293 studies from PubMed, EMBASE, the Cochrane Library, CNKI, CBM and WanFang Data, and other resources. A total of 196 studies remained after removing duplicate articles. Titles and abstracts of all recognized studies were reviewed to isolate the relevant articles. We read the entire text of a total of 37 potentially relevant articles. Among these, 28 articles were excluded for the following reasons: Six studies included a duplicated study population; 15 studies suffered from insufficient data; 4 studies included subjects who received only a partial pathological examination; 2 studies did not use the 2003 WHO Classification of Tumors of the Breast and Female Genital Organs^[11]^; 1 study was not published. The remaining nine studies were selected for our meta-analysis.^[7,16–23]^

### 3.2 Characteristics and quality assessment of selected studies

**Figure 2:**
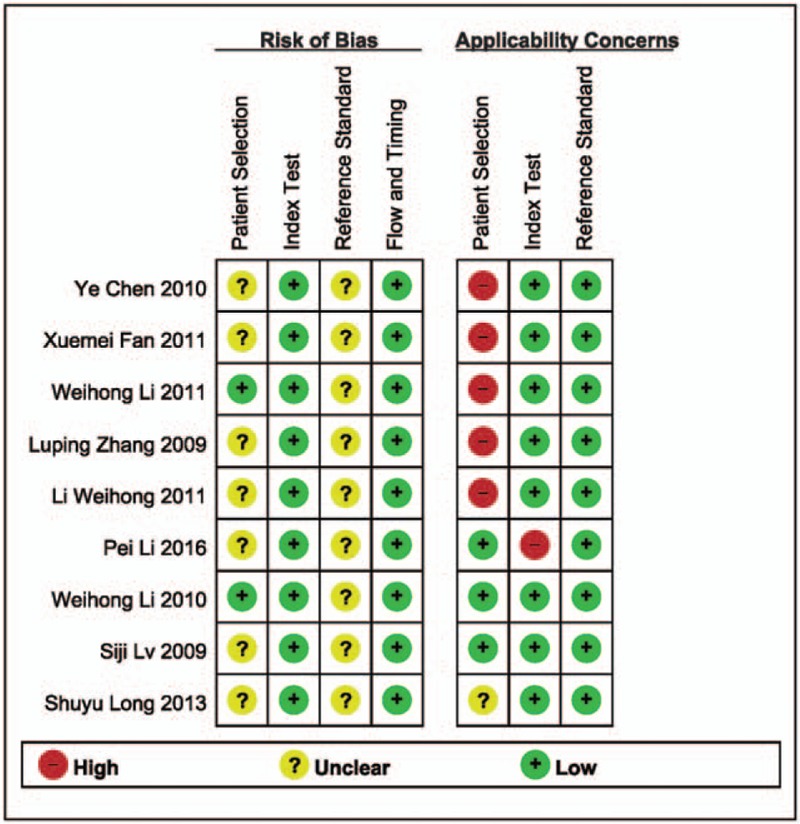
Summary of the methodological assessment of the included studies basing on the Cochrane Handbook. +: Low risk; –: High risk; ?: Unclear.

Table 1 displays the principle characteristics of included studies. The nine studies included a total of 2730 patients. The age ranged from 20 to 65 years. Two studies were conducted across multiple centers.^[16,22]^ The remaining were single-center studies.^[7,17–21,23]^ All studies were conducted in various regions of China from 2009 to 2016. Five studies enrolled patients with cervical cytology positive results; three studies enrolled patients in gynecological clinics with cervical lesions; one study enrolled patients who consented to cervical loop electrosurgical excision procedure surgery. A summary of the methodological assessment based on QUADAS-2 is shown in Figure 2. All nine studies reported the range of study time;^[7,16–23]^ Two studies reported that patients were recruited randomly;^[18,22]^ the remaining 7 studies did not indicate whether patients were recruited consecutively or randomly.^[7,16,17,19–21,23]^ Seven studies reported that the index test was always conducted and interpreted prior to performance of reference standard test.^[7,17–20,22,23]^ Two studies specified that results of the index test and the reference standard test were collected on the same patients at the same time;^[7,17]^ the remaining 7 studies failed to report the interval between the index test and the reference standard test.^[16,18–23]^ Patients in all 9 studies underwent the reference standard test. All were included in the analysis.^[7,16–23]^

**Table 1:**
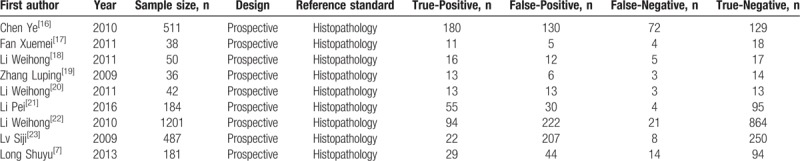
Characteristics of the included studies.

### 3.3 Overall meta-analysis

**Figure 3:**
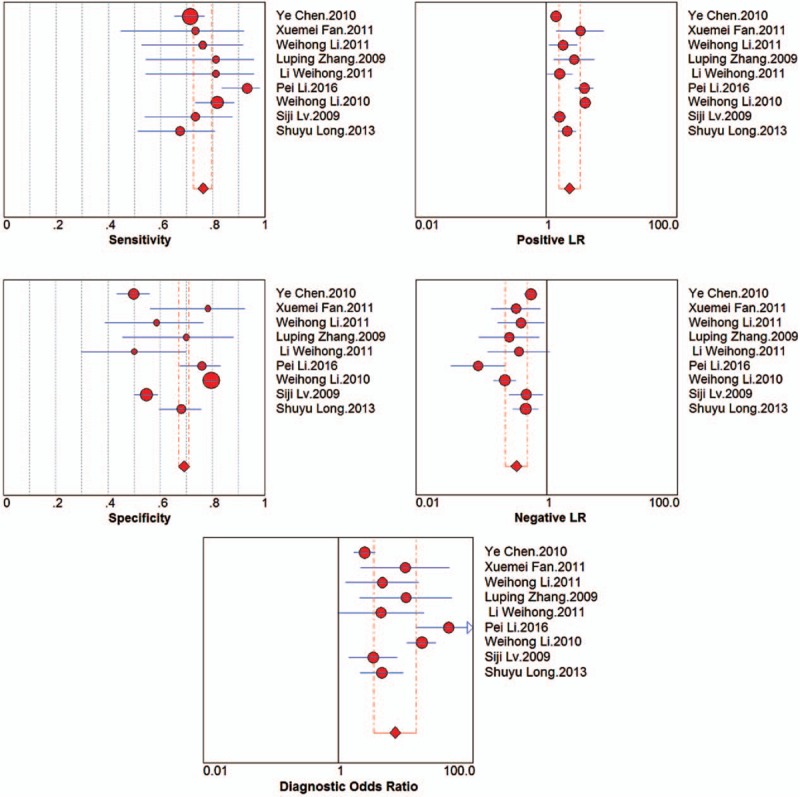
Diagnostic analysis of the TruScreen for cervical cancer screening.

**Figure 4:**
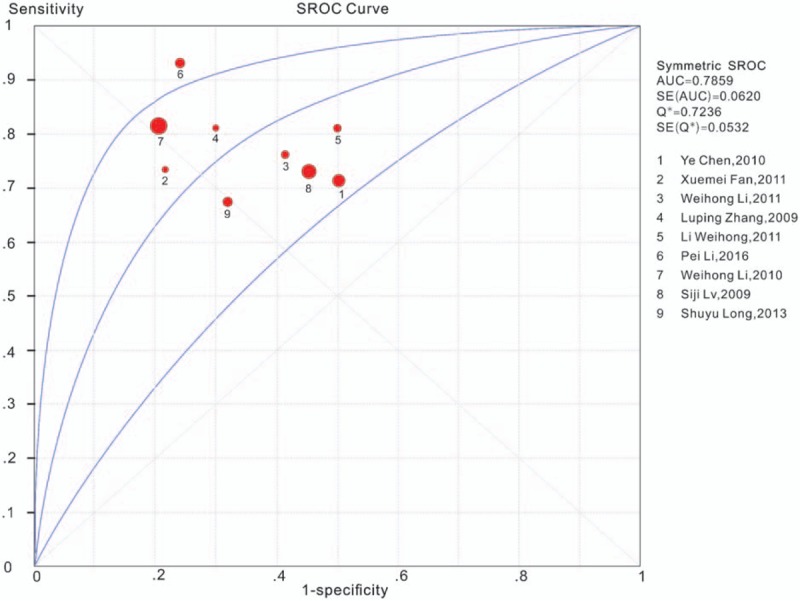
Summary receiver operating characteristic (SROC) curve of the TruScreen for cervical cancer screening.

For the TruScreen, the pooled sensitivity, pooled specificity, PLR and NLR were 76% (95% CI, 73%–80%), 69% (95% CI, 67%–71%), 2.30 (95% CI, 1.59–3.33), and 0.34 (95% CI, 0.23–0.51), respectively; the DOR was 7.03 (95% CI, 3.40–14.55). Figure 3 shows the diagnostic analysis of the TruScreen. The SROC curves for the TruScreen device are displayed in Figure 4. The AUC was 0.7859 (*Q*^∗^ = 0.7236), indicating moderate accuracy. A bivariate model was used in this meta-analysis. A moderate degree of heterogeneity was detected (*Q* = 4.096, df = 2, *P* = .064, *I*^2^ = 51% [95% CI, 0–100]).

### 3.4 Meta-regression

To locate the source of the heterogeneity, we conducted a meta-regression analysis based on patient type (patients in gynecological clinics whose cervical cytology may be normal, or patients with positive cervical cytology who planned to undergo colposcopy). Sensitivity and specificity according to patient type demonstrated a statistically significant difference (*P* < .05): 79% (95% CI, 72%–86%) and 71% (95% CI, 62%–79%) in studies enrolling patients whose cervical cytology result may be normal, versus 76% (95% CI, 69%–84%) and 60% (95% CI, 48%–71%) in studies enrolling patients with positive cervical cytology results.

### 3.5 Publication bias

**Figure 5:**
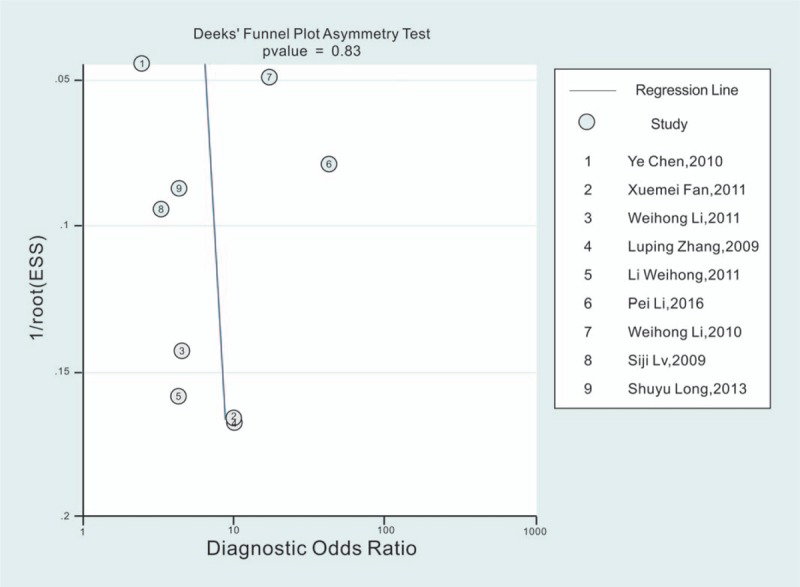
Deek's funnel plot for the assessment of potential publication bias of the included studies.

We used the Deeks funnel plot to assess the publication bias. As shown in Figure 5. The slope coefficient did not reveal obvious evidence of asymmetry, with a *P*-value of .832, suggesting a low probability of publication bias.

## 4 Discussion

Technological advances in cervical cancer screening have developed rapidly over time. Cervical cancer screening technologies primarily consist of cytologic tests, HPV DNA tests and visual inspection. The ThinPrep cytologic test (TCT), the HPV test, and the combined test (TCT & HPV) all have high sensitivity and specificity.^[24]^ However, these tests are expensive, require a high level of technological skill, and entail long waiting times to obtain results. Therefore, such tests may not be suitable for widespread use in the developing world. The WHO recommended visual inspection as the cervical cancer screening method of choice in low-resource areas.^[25]^ Unfortunately, visual inspection is highly subjective and quality control is difficult. According to a survey carried out in India and Africa by IARC, the variation of sensitivity and specificity for visual inspection was 56.1% to 93.9% and 74.2% to 93.8%, respectively.^[26]^ To reduce the incidence of cervical cancer in low-resource areas, it is necessary to identify a screening method that is simple, affordable, non-invasive, instant, and can increase screening coverage. The test must also be objective and possess adequate sensitivity and specificity.

According to our meta-analysis, we found that the pooled sensitivity and pooled specificity of the TruScreen is 76% and 69% respectively. The AUC of TruScreen was 0.7859 (*Q*^∗^ = 0.7236). This suggests that the device is moderately accurate. Meanwhile, this device is noninvasive, simple to operate, may be performed by nurses or assistants after training, and the test results could be available immediately and objectively. Therefore, this device could be operated in primary medical institutions and regions lacking medical staffs. In addition, this device is low-cost for screening, employing single-use sensors, thus enabling wide use for cervical cancer screening in economically backward areas. However, this device has 2 disadvantages: one disadvantage is that the specificity of the device is relatively low and the false positive rate is high, possibly leading to nonessential inspection and psychological burden on patients; the other disadvantage is that the device is not good at detecting cervical canal lesions. To increase the specificity, sensitivity and the clinical value of the TruScreen, it is recommended that the use of this device be combined with other cervical cancer screening methods. If it were used alone, it would not be recommended for applying to postmenopausal women in whom the squamous-columnar junction of the cervix had moved up into cervical canal.

The studies included in our analysis were moderately homogenous (*P* = .064, *I*^2^ = 51%). This finding suggests that differences among studies probably affect the results of our meta-analysis. In order to investigate possible cause of this heterogeneity, we performed a meta-regression analysis. We found that patient type was a statistically significant factor affecting heterogeneity (*P* < .05). The TruScreen device has a higher sensitivity and specificity when studies involved patients in gynecological clinics, as opposed to studies involved patients with positive cervical cytology results who planned to undergo colposcopy. This finding may be explained by selection bias and/or spectrum bias. Together, our results suggest that the TruScreen was suitable for universal screening, especially for the low-resource areas with high incidence of cervical cancer.

The findings of our meta-analysis should be interpreted in light of the following limitations. First, we only included 9 studies, all of which were conducted in China. Second, due to the limited number of patients, we did not analyze the performance of the TruScreen device for detection of CIN II or higher. Third, we did not include “gray” literature, unpublished studies, or research abstracts from meeting proceedings. Such studies are not commonly subjected to peer-review and they provided limited data. However, by choosing to eliminate these studies, we might have overlooked potentially relevant studies. Fourth, data from the nine studies were collected in advanced areas and hospitals where there were well-trained medical doctors. If the study were conducted in remote mountain/village areas by a part-time medical assistant, the conclusions may have been different.

In the process of carrying out our meta-analysis, we found that some studies only perform the gold standard test on patients with a positive cervical cytology result or a positive index test result. This may lead to partial verification bias or work-up bias. Future researchers should remain alert to this kind of bias.

Our meta-analysis only evaluated the performance of the TruScreen device from the point of view of statistics, not economics. We expect that future investigators will evaluate cost-effectiveness, cost-utility and cost-benefit ratios of the device.

In conclusion, the diagnostic value of this real-time optoelectronic device is moderate at best. Given that the number of included studies in the meta-analysis is relatively small and all studies were conducted in China, therefore the study findings could not be generalized to other populations and should be interpreted with caution.

## Author contributions

**Conceptualization:** Huixia Yang, Zengping Hao.

**Data curation:** Huixia Yang, Zengping Hao, Xinmiao Zhang.

**Formal analysis:** Huixia Yang, Zengping Hao, Xinmiao Zhang.

**Funding acquisition:** Huixia Yang, Zengping Hao, Xinmiao Zhang.

**Investigation:** Huixia Yang, Zengping Hao, Xinmiao Zhang.

**Methodology:** Huixia Yang, Zengping Hao, Xinmiao Zhang.

**Project administration:** Huixia Yang, Zengping Hao, Xinmiao Zhang.

**Resources:** Huixia Yang, Zengping Hao, Xinmiao Zhang.

**Software:** Huixia Yang, Zengping Hao, Xinmiao Zhang.

**Supervision:** Huixia Yang, Zengping Hao, Xinmiao Zhang.

**Validation:** Huixia Yang, Zengping Hao, Xinmiao Zhang.

**Visualization:** Huixia Yang, Zengping Hao, Xinmiao Zhang.

**Writing – original draft:** Huixia Yang.

**Writing – review & editing:** Zengping Hao.

## References

[1] Ferlay J Soerjomataram I Ervik M Dikshit R Eser S Mathers C Rebelo M Parkin DM Forman D Bray F GLOBOCAN 2012 v1.0, Cancer Incidence and Mortality Worldwide: IARC CancerBase No. 11 [Internet]. Lyon, France: International Agency for Research on Cancer; 2013 Available at: http://globocan.iarc.fr Accessed on March 4, 2018.

[2] Arbyn M Castellsagué X Sanjosé SD Worldwide burden of cervical cancer in 2008. Ann Oncol 2011;22:2675–86. 21471563 10.1093/annonc/mdr015

[3] Andrae B Kemetli L Sparén P Screening-preventable cervical cancer risks: evidence from a nationwide audit in Sweden. J Natl Cancer Inst 2008;100:622–9. 18445828 10.1093/jnci/djn099

[4] World Health Organization. WHO guidelines for screening and treatment of precancerous lesions for cervical cancer prevention: supplemental material: GRADE evidence-to-recommendation tables and evidence profiles for each recommendation. World Health Organization 2013.

[5] Jordan JA Singer A The cervix. Second Edition[M] 2009;387–94.

[6] Singer A Coppleson M Canfell K A real time optoelectronic device as an adjunct to the Pap smear for cervical screening: a multicenter evaluation. Int J Gynecol Cancer 2003;13:804. 14675317 10.1111/j.1525-1438.2003.13393.x

[7] Long S Lei W Feng Y The feasibilities of TruScreen for primary cervical cancer screening: a self-controlled study. Arch Gynecol Obstet 2013;288:113. 23296464 10.1007/s00404-012-2697-4

[8] Özgü E Yildiz Y Özgü BS Efficacy of a real time optoelectronic device (TruScreen) in detecting cervical intraepithelial pathologies: a prospective observational study. J Turk Ger Gynecol Assoc 2015;16:41–4. 25788849 10.5152/jtgga.2015.15199 PMC4358311

[9] Moher D Liberati A Tetzlaff J The PRISMA Group (2009). Preferred reporting items for systematic reviews and meta-analyses: the PRISMA statement. BMJ 2009;339:b2535. 19622551 10.1136/bmj.b2535 PMC2714657

[10] Bossuyt PM Leeflang MM Chapter 6: Developing Criteria for Including Studies. In: Cochrane Handbook for Systematic Reviews of Diagnostic Test Accuracy Version 0.4 [updated September 2008]. The Cochrane Collaboration, 2008.

[11] IARC Sci Publ, Tavassoli FA Devilee P World Health Organization. Pathology and Genetics of Tumours of the Breast and Female Genital Organs[M]. 2003;398–399.

[12] Whiting PF Rutjes AW Westwood ME QUADAS-2: a revised tool for the quality assessment of diagnostic accuracy studies. Ann Intern Med 2011;155:529–36. 22007046 10.7326/0003-4819-155-8-201110180-00009

[13] Swets JA Measuring the accuracy of diagnostic systems. Science 1988;240:1285–93. 3287615 10.1126/science.3287615

[14] Walter SD Properties of the summary receiver operating characteristic (SROC) curve for diagnostic test data. Stat Med 2002;21:1237–56. 12111876 10.1002/sim.1099

[15] Higgins JP Thompson SG Quantifying heterogeneity in a meta-analysis. Stat Med 2002;21:1539. 12111919 10.1002/sim.1186

[16] Chen Y Multicenter research on the comparison between TruScreen Cervical Cancer system and liquid—based cytological test in the screening of cervical lesions. (Doctoral dissertation, Sun Yat-sen University in Guangzhou, China, 2010).

[17] Fan X Zhang Q Guo Z Application study about TruScreen on screen of cervical lesions. Guangzh Med J 2011;42:37–9.

[18] Li W Ling Y Zhang J Value of different target area in TruScreen system for the screening of cervical cancer. J Hainan Med Univ 2011;17:1171–4.

[19] Zhang L Zhang S Li W The performance of Truscreen in the patients with the cytologic diagnosis of epithelial cell abnormality. China Healthcare Frontiers 2009;04:62–3.

[20] Li W Guo Y Niu H Application of TruScreen in detecting ASCUS patients. Asian Pac J Trop Med 2011;4:669–71. 21914550 10.1016/S1995-7645(11)60170-3

[21] Li P Wang J Kang Y Application effect of TruScreen system in cervical cancer screening. Oncol Progr 2016;14:557–8.

[22] Li WH Jin S Zhang JX Efficacy comparison of cervical cancer screening system and liquid based cytology test in detection of cervical lesions. J Hainan Med Univ 2010;16:1129–31.

[23] Lv S Huang L Xin Z Comparing study of cervical cancer screening system and liquid-based cytology test in the screening of cervical lesions. Progr Obstet Gynecol 2009;18:90–3.

[24] De SS Ibáñez R Rodríguezsalés V Screening of cervical cancer in Catalonia 2006-2012. Ecancermedicalscience 2015;9:532. 25987901 10.3332/ecancer.2015.532 PMC4431403

[25] World Health Organization. IARC handbooks of cancer prevention. Volume 10: Cervix cancer screening.[M] 2005.

[26] Sankaranarayanan R Basu P Wesley RS Accuracy of visual screening for cervical neoplasia: Results from an IARC multicentre study in India and Africa. Int J Cancer 2004;110:907–13. 15170675 10.1002/ijc.20190

